# Mapping the Gaps: A Scoping Review of Virtual Care Solutions for Caregivers of Children with Chronic Illnesses

**DOI:** 10.3390/children12010077

**Published:** 2025-01-10

**Authors:** Nicole Pope, Kathyrn A. Birnie, Melanie Noel, Justine Dol, Danyu Li, Megan Macneil, Darrel Zientek, Victoria Surry, Jennifer N. Stinson

**Affiliations:** 1 Child Health Evaluative Services, Research Institute, The Hospital for Sick Children, Toronto, ON M5G 1E8, Canada; jennifer.stinson@sickkids.ca; 2 Department of Nursing, Melbourne School of Health Sciences, Faculty of Medicine, Dentistry and Health Sciences, The University of Melbourne, Melbourne, VIC 3052, Australia; 3 Murdoch Children’s Research Institute, Melbourne, VIC 3052, Australia; 4 Department of Psychology and Neuroscience, Faculty of Science, Dalhousie University, Halifax, NS B3H 4R2, Canada; kathryn.birnie@ucalgary.ca; 5 Department of Anesthesiology, Perioperative and Pain Medicine, University of Calgary, Calgary, AB T2N 1N4, Canada; 6 Department of Community Health Sciences, University of Calgary, Calgary, AB T2N 1N4, Canada; 7 Department of Psychology, University of Calgary, Calgary, AB T2N 1N4, Canada; melanie.noel@ucalgary.ca; 8 Alberta Children’s Hospital Research Institute, Hotchkiss Brain Institute, Calgary, AB T2N 4N1, Canada; 9 Centre for Pediatric Pain Research, IWK Health, Halifax, NS B3K 6R8, Canada; justine.dol@dal.ca; 10 Shanghai Institute of Infectious Disease and Biosecurity, Fudan University, Shanghai 200433, China; danyuli365@outlook.com; 11 School of Nursing, Fudan University, Shanghai 200433, China; 12 Chronic Pain Network, McMaster University, Hamilton, ON L8S 4L8, Canada; megan.macneil@ucalgary.ca; 13 School of Public Health, University of Alberta, Edmonton, AB T6G 2R3, Canada; 14 Independent Researcher, Ottawa, ON K2J 4W5, Canada; lcolzman@gmail.com; 15 Faculty of Human and Social Development, School of Public Administration, University of Victoria, Victoria, BC V8P 5C2, Canada; victoriasurry@uvic.ca; 16 Lawrence S. Bloomberg Faculty of Nursing and IHPME, University of Toronto, Toronto, ON M5S 1A1, Canada

**Keywords:** virtual care, chronic illnesses, chronic pain, children, adolescents, primary caregivers, evidence and gap map, stepped care

## Abstract

**Background/Objectives**: Caregivers of children with chronic illnesses, including chronic pain, experience high levels of distress, which impacts their own mental and physical health as well as child outcomes. Virtual care solutions offer opportunities to provide accessible support, yet most overlook caregivers’ needs. We conducted a scoping review to create an interactive Evidence and Gap Map (EGM) of virtual care solutions across a stepped care continuum (i.e., from self-directed to specialized care) for caregivers of youth with chronic illnesses. **Methods**: The review methodology was co-designed with four caregivers. Data sources were the peer-reviewed scientific literature and a call for innovations. Records were independently coded and assessed for quality. **Results**: Overall, 73 studies were included. Most virtual care solutions targeted caregivers of children with cancer, neurological disorders, and complex chronic illnesses. Over half were noted at lower levels of stepped care (i.e., self-guided apps and websites), with psychological strategies being predominant (84%). However, very few addressed caregivers’ physical health (15%) or provided family counseling (19%) or practical support (1%). Significant gaps were noted in interventions for managing caregiver chronic pain, despite its high prevalence and impact on child outcomes. **Conclusions**: Evidence and Gap Maps are innovative visual tools for knowledge synthesis, facilitating rapid, evidence-informed decision-making for patients, families, health professionals, and policymakers. This EGM highlighted high-quality virtual care solutions ready for immediate scaling and identified critical evidence gaps requiring prioritization. To address the complexities of pediatric chronic illnesses, including chronic pain, virtual care initiatives must prioritize family-centered, accessible, and equitable approaches. Engaging caregivers as partners is critical to ensure interventions align with their needs and priorities.

## 1. Introduction

Chronic pain is a public health crisis affecting one in five children (aged 5–12 years) and adolescents (aged 13–18 years) worldwide [1]. Pediatric chronic pain often co-exists with other chronic health issues. It is associated with profound costs (CAD 40 billion direct and indirect costs) [2], disability, and devastating impacts on the mental health of youth and their families [3]. Primary caregivers (i.e., parents, hereafter ‘caregivers’), particularly those from minoritized and marginalized backgrounds, are at increased risk of mental health problems [4,5], disrupted sleep, and reduced overall well-being due to caregiving responsibilities, emotional distress [4,5,6,7], and socioeconomic strain [8]. These challenges are shared across different chronic conditions [9].

Caregivers are critical to optimal health outcomes for youth with chronic illnesses, including chronic pain, yet they receive very little support [6]. They suffer high levels of chronic stress and poor physical and mental health [4,5,6,7,8,9], and challenges in parenting, relationships, and their professional lives [8,9,10]. Poor caregiver well-being and the absence of supportive relationships strongly predict adverse clinical (physical, psychological, and developmental) child outcomes [11,12]. For example, high levels of caregiver distress impede children’s response to psychological chronic pain interventions [13]. Caregiver mental health issues, such as anxiety and stress, are powerful predictors of worse pain outcomes in children [5,13]. Although caregiver-focused interventions have demonstrated benefits [14], they most often target parenting behaviors, such as reducing over-protective responses to child pain [15,16], rather than directly addressing caregiver mental health (e.g., cognitions and emotions) and their own chronic pain. Caregiver chronic pain strongly influences child outcomes through mechanisms such as modeling, reinforcement, shared genetics, and environmental factors [17,18,19]. Addressing caregiver mental health and chronic pain directly is critical to mitigate the rippling effects on youth, families, and society.

Early interventions for pediatric chronic illness, including chronic pain, reduce progression, severity, disability, mental health issues [20,21,22], substance use, and healthcare costs [23,24]. Yet most children and youth with chronic pain and their families never receive this care [25], a problem amplified by the COVID-19 pandemic [26,27]. Marginalized and minoritized families often face inequities in healthcare access [28,29,30], stigmatization [31,32], systemic barriers (e.g., racism) [33,34,35], and socioeconomic factors that negatively impact caregiver and child mental health across chronic conditions [7,36].

**Figure 1:**
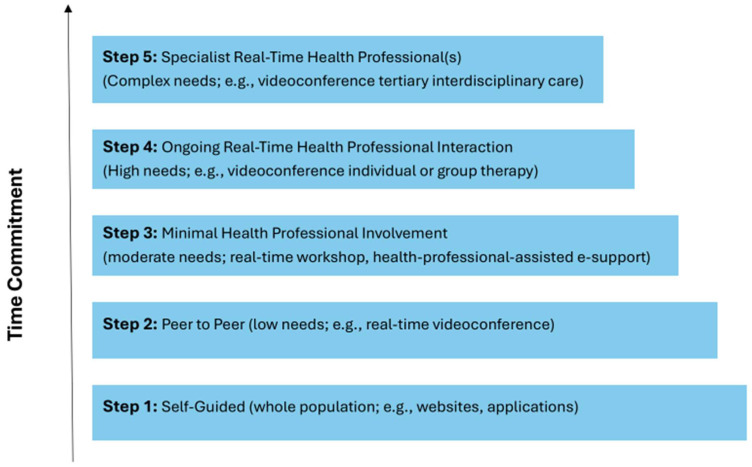
Levels of stepped care. Model adapted from Mental Health Commission of Canada, *Newfoundland and Labrador Stepped Care 2.0 e-Mental Health Demonstration Project*: Health Canada: 2019 [38].

Virtual care solutions offer a critical opportunity to bridge these gaps in access by providing flexible, scalable, and inclusive resources for diverse families of children with chronic illnesses [21], including those in underserved communities [37]. Virtual care includes remote therapies accessed by patients or families or delivered by healthcare providers through telehealth, mobile apps, websites, text messaging, and videoconferencing [36,37]. These solutions help overcome access barriers (i.e., geographic distance, financial burden, scarcity of local specialists) [37,38]. They can be tailored depending on the severity of symptoms and disability, following a stepped care continuum [38,39] (see Figure 1). This approach ranges from self-directed strategies to manage symptoms (low involvement, low resource) to ongoing specialist consultations (high involvement, high resource) [38,39].

This scoping review takes a novel and inclusive approach by integrating the stepped care framework into caregiver interventions and using an Evidence and Gap Map (EGM) to categorize and visualize virtual care solutions. By expanding its focus beyond chronic pain to include virtual care interventions for caregivers of children with a range of chronic illnesses, the review identifies shared challenges and opportunities across diverse conditions. This broader perspective builds on prior research and provides a powerful tool to identify gaps and prioritize interventions that address diverse caregiver needs.

A recent review incorporated peer-reviewed and grey literature, and a call for virtual care innovations to identify virtual care solutions for youth with chronic pain and their parents and highlighted areas for further development [40]. Among the 185 studies included, most focused on lower levels of stepped care (e.g., over 100 self-guided apps and websites) and commonly used virtual psychological strategies [40]. Significant evidence gaps were identified in higher levels of stepped care and content addressing school, medications, acute pain crises, and primary caregiver mental health and chronic pain [40].

These gaps highlight the lack of tailored interventions to support caregivers’ mental and physical health, including managing their own pain, which affects up to 50% of caregivers in tertiary pediatric pain clinics [5]. Addressing this issue is crucial, as the pandemic’s long-term effects could worsen disability and the suffering of children and caregivers [24,25,40]. To provide a broader perspective, the scope of this scoping review has been expanded to include virtual care interventions for caregivers of children with chronic illnesses, not just chronic pain. By examining broader interventions, shared challenges across chronic conditions could be identified.

This review aimed to map and summarize the literature on virtual care solutions, addressing the physical, mental, and emotional health of caregivers of children (0–18 years) with chronic illness. By introducing the EGM as a tool, this review highlights the potential to bridge evidence gaps, guide intervention development, and prioritize areas of unmet need in a structured and actionable manner. The resulting EGM categorized solutions across levels of stepped care and served as a visual tool to inform evidence-informed decision-making for patients, families, health professionals and policymakers [40,41,42]. To ensure a patient-centered focus, caregivers of youth with chronic pain were involved in identifying the most relevant components of virtual care solutions.

## 2. Materials and Methods

### 2.1. Patient Engagement

The project team conducting the scoping review included three caregivers of youth with lived experience of chronic pain as patient partners(M.M., D.Z., V.S.), pediatric pain health professionals (N.P., K.A.B., J.S., M.N., J.D., E.T., G.E., D.L.) and researchers (N.P., K.A.B., J.S., M.N.), and medical and nursing graduate and post-doctoral trainees (J.D., E.T., G.E., D.L.). We followed the GRIPP-2 reporting guidelines for patient and public involvement in research [43]. Caregiver partners were engaged as full collaborators [44,45,46] and compensated following best practices to acknowledge their expertise and contributions [46,47]. They were involved in co-designing the content and language of the EGM and contributed to co-authorship.

### 2.2. Protocol and Registration

The Joanna Briggs Institute Manual for Evidence Synthesis for scoping reviews guided the development of the protocol [48], methods recommended by Arksey and O’Malley [49], and other recommendations for the conduct of scoping reviews were also used [50]. This scoping review was conducted using two sources of information: the scientific literature and a call for innovations. The results were subsequently synthesized in an EGM. Evidence and Gap Maps are an interactive data synthesis tool that showcases the breadth, availability, and quality of evidence within a specific field [41,42]. They offer an innovative knowledge synthesis approach to accelerate the adoption of virtual care solutions for caregivers of youth with chronic pain. The EGM visual summary delivers an accessible and practical synthesis of evidence, highlighting strengths and gaps to guide decisions in practice, policy, research, and investment [40,41,42].

The PRISMA Extension for Scoping Reviews guidelines [51] were followed for quality reporting at each review phase. The review protocol was pre-registered and published in the Open Science Framework registries (https://archive.org/details/osf-registrations-5pefu-v1, accessed on 18 December 2024).

### 2.3. Eligibility Criteria, Search Strategy, and Conduct

#### 2.3.1. Scientific Literature Search

The inclusion and exclusion criteria were defined following the PCC (P, population; C, concept; C, context) criteria [48]. English peer-reviewed scientific studies published in the past 10 years reporting original research using any methodology were identified for inclusion if they (1) discussed caregivers (i.e., parents, grandparents) of children and adolescents (aged 0 to 18 years) with chronic illnesses, (2) focused on any type of virtual care solution (e.g., telephone, telehealth, telemedicine, mHealth, eHealth, online, and digital), and (3) were primary studies of any type with an identified purpose of evaluating virtual care solutions (e.g., randomized controlled trials [RCTs], nonrandomized trials, observational studies, mixed-methods studies, qualitative studies, and case reports). Pilot studies or trials of the intervention were considered if they reported caregiver outcomes (see below). Studies that described the intervention with no pilot testing data were excluded. Studies focused only on usability testing, intervention development, or child-related outcomes only were omitted. In this review, virtual care within a stepped care continuum was defined as any caregiver-targeted intervention delivered or accessed entirely remotely through any form of communication or information technologies. The five levels of the stepped care continuum and examples are presented in Figure 1, adapted from the Mental Health Commission of Canada’s Stepped Care 2.0 model [38]. Studies were excluded if they required any in-person component. Systematic reviews were excluded to avoid duplication of evidence and to maintain a clear focus on the review’s objectives. This exclusion enabled a more comprehensive mapping of original studies and emerging areas, supporting the identification of gaps for future research.

#### 2.3.2. Search Strategy

The search strategy was developed with a health sciences librarian and the research team. The MEDLINE (via Ovid), EMBASE (via Ovid), CINAHL (via EBESCO), PsycINFO, and PubMed databases were electronically searched between 22 December 2023 and 4 January 2024. The database-specific search strings are shown in the supplemental digital content [SC T1]. Additional records were identified by an additional citation search from the reference lists of included studies (“hand/citation search”). To maintain a manageable scope, we did not include grey literature (i.e., dissertations, commentaries, conference abstracts).

#### 2.3.3. Call for Innovations

To ensure our review remained current and inclusive, we issued a call for demonstrated and emerging virtual care innovations to capture novel developments or initiatives that may have not yet been represented in the academic literature and/or were under empirical investigation. The call focused on interventions that support the mental and physical health and parenting needs of caregivers of children (aged 18 years and younger) living with chronic illness (including but not limited to chronic pain) and followed a methodology previously used by co-authors to identify innovations for youth with chronic pain [40]. The online call was launched in March 2024 and remained open until June 2024.

Eligible applicants included those from healthcare organizations (public and private) and community, government, and social service sectors. Applicants completed an online application, which included a description of the virtual care innovation, its application to caregivers of children with chronic pain, its focus on addressing the mental, physical health, and parenting needs of caregivers of children living with chronic illness (including but not limited to chronic pain), and any completed or ongoing evaluation. The call was emailed to pediatric chronic pain programs in North America and Australia, Listservs (Society of Pediatric Psychology, Pediatric Pain, and Pain in Child Health), and partner organizations (Melbourne Children’s Campus).

The call prioritized chronic pain programs due to their direct relevance to the study focus while remaining inclusive of caregivers of children with broader chronic illnesses to capture intersecting needs. Targeting well-networked sectors, such as specialized Listservs and partner organizations, ensured access to practitioners and researchers with relevant expertise while maintaining a manageable scope to facilitate coordination and evaluation.

#### 2.3.4. Screening of Studies, Data Coding, and Quality Appraisal

All identified studies were screened after the exclusion of duplicates using titles and abstracts by eight independent reviewers (N.P., S.O., F.M., A.N., E.T., B.M., D.L., J.S.) in a double-blinded manner by use of the Covidence program (Veritas Health Innovation) [52]. The same eight reviewers undertook subsequent full-text screening; at least two reviewers reviewed every abstract and full text. All disagreements were resolved through consensus and/or consultation with another project team member (J.S., K.B.). To ensure consistency and quality, we facilitated training sessions to familiarize reviewers with the screening criteria, Covidence, and the double-blinded process. Clear guidelines and documentation were provided to standardize the approach, and regular consensus meetings were held to address challenges, clarify criteria, and resolve uncertainties.

Next, data extraction was conducted by four reviewers from the same team (N.P., E.T., D.L., J.D.). To assess and ensure intercoder reliability, we conducted pilot testing during the data extraction phase, achieving a high agreement rate (>85%) among reviewers. Differences in extraction were discussed, and the result was the refinement of extraction criteria applied by all reviewers on all extractions. Furthermore, regular consensus meetings were held to address any discrepancies, refine extraction criteria, and ensure a consistent and reliable approach across all reviewers. One reviewer extracted the remaining records; a second independent reviewer reviewed a 40% random sample. There were no disagreements between reviewers. Authors of the included papers were contacted to request missing or additional information.

The following data were gathered from included studies: (1) study characteristics (first author, publication year, funding, country of study conduction, study design, description of study population including disease and age of caregivers and children), (2) information on interventions (goal(s) of intervention, intervention targets, and components of virtual care solutions identified), (3) all outcome measures, (4) technology platform (e.g., videoconference, telephone, and smartphone), and (5) relevance to level of stepped care [38].

All records were independently evaluated for quality using the Mixed Methods Appraisal Tool (MMAT-v2018) as it is applicable across various study types, including qualitative, quantitative RCT, quantitative nonrandomized, quantitative descriptive, and mixed-methods research [53,54]. The MMAT consists of two methodological screening questions applicable to all study types and five specific methodological criteria questions tailored to each study type. Responses to each question were categorized as “yes”, “no”, or “cannot tell”. Although the MMAT does not generate a single quality summary score, the research team developed a scoring system to categorize the quality of scientific evidence for the interactive EGM as high, moderate, low, or critically low. Studies were rated as high quality if they met 4 to 5 of the methodological quality criteria specified for their respective study type (with “yes” responses), moderate if they met 2 to 3 criteria, low if they met 1 criterion, and critically low if they met none. A study was deemed not to meet a criterion if it explicitly reported not meeting it or if the information necessary to evaluate the criterion was unavailable in the article (indicated by a “cannot tell” response).

## 3. Results

### 3.1. Study Selection

**Figure 2:**
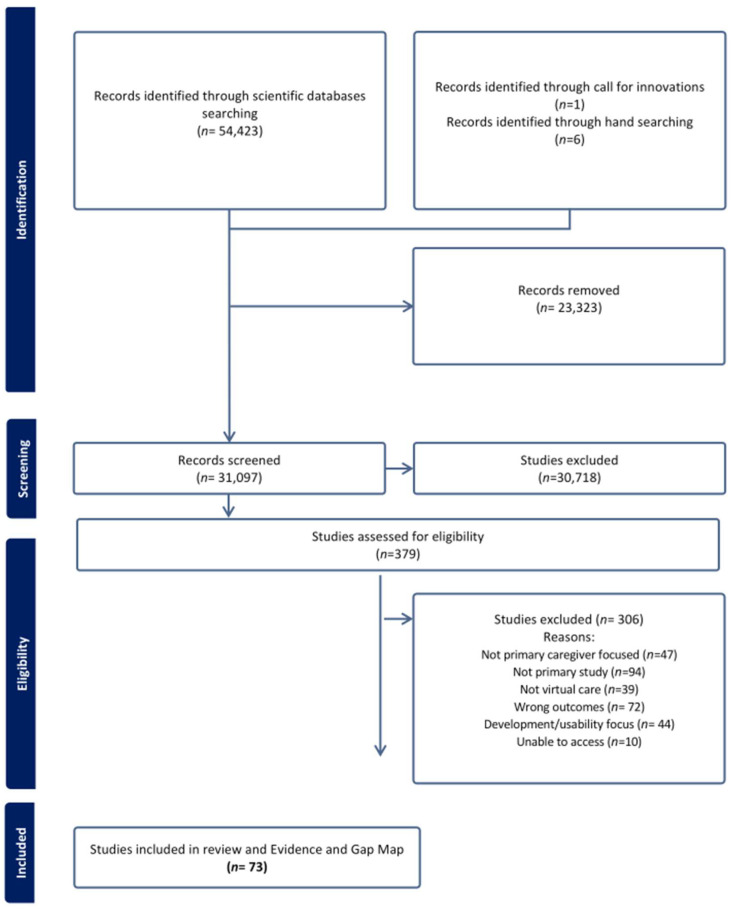
PRISMA flow diagram.

Scientific database searches identified 54,423 records. In addition, six studies were identified by hand/citation searching. After removing all duplicates, 31,097 abstracts were screened according to the pre-defined inclusion and exclusion criteria. After resolving disagreements, 379 full-text studies were assessed for eligibility. Finally, 73 studies met the inclusion criteria and were included in the scoping review. Only one of these was identified through the issued call for innovations. See Figure 2 for the PRISMA review flowchart with reasons for exclusion.

### 3.2. Study Characteristics

Given the number of records that fulfilled the review’s inclusion criteria and the open-access availability of these records and their corresponding codes in the interactive EGM, a complete list of references for all records is provided as Supplementary Material [SC T2] (and is accessible at https://lab.research.sickkids.ca/iouch/caregivers/, accessed on 18 December 2024) instead of citing each reference repeatedly throughout the results summary. Each record included in the EGM features its title, a summary description, authorship, publication year, URL, and scientific publication details (such as journal name, volume, issue, page numbers, and DOI). Of the 73 studies meeting the inclusion criteria, the most frequent study type was RCTs (61.6%, *n* = 45), followed by observational studies (20.5%, *n* = 15), mixed-methods studies (11.0%, *n* = 8), qualitative studies (5.5%, *n* = 4), and a case series study (1.4%, *n* = 1).

### 3.3. Types of Populations

Of the virtual care solutions, 26% (*n* = 19) targeted caregivers of children with cancer, 17.8% (*n* = 13) included caregivers of children with neurological conditions (e.g., traumatic brain injury), 13.7% (*n* = 10) focused on caregivers of children with ‘chronic/complex illness’, and 11.1% (*n* = 8) included caregivers of children with chronic pain. The rest of the records included caregivers of children with developmental conditions (e.g., Autism Spectrum Disorder [ASD], cerebral palsy, intellectual disabilities) (9.6%, *n* = 7), learning and attentional conditions (i.e., Attention Deficit Hyperactivity Disorder [ADHD]) (5.4%, *n* = 4), and other chronic conditions including type 1 diabetes (5.4%, *n* = 4), chronic kidney disease (2.7%, *n* = 2), chronic respiratory disease (2.7%, *n* = 2), gastrointestinal illness (e.g., inflammatory bowel disease [IBD]) (1.4%, *n* = 1), coagulation factor disorder (1.4%, *n* = 1), eating disorders (1.4%, *n* = 1), and mental health conditions (1.4%, *n* = 1).

### 3.4. Level of Stepped Care

**Figure 3:**
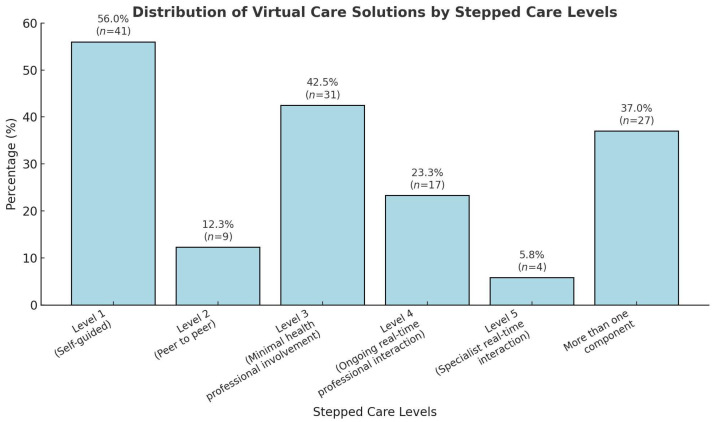
Virtual care solutions by stepped care levels.

Over half of the identified virtual care solutions had a self-guided component (56%, *n* = 41) (i.e., level 1 stepped care), 42.5% (*n* = 31) had a component classified as requiring minimal health professional involvement (i.e., level 3 of stepped care) and 23.3% (*n* = 17) had a component that required ongoing real-time health professional interaction (i.e., level 4 of stepped care). The rest of the solutions contained a peer-to-peer element (i.e., level 2 of stepped care) (12.3%, *n* = 9) or involved specialist real-time health professional interaction (level 5 of stepped care) (5.8%, *n* = 4). Thirty-seven percent of the solutions (*n* = 27) comprised more than one component (see Figure 3).

Figure legend footnote: The stepped care model categorizes virtual care solutions by the level of health professional involvement required. Level 1 (Self-guided): Interventions that are independent and do not require health professional involvement (e.g., self-help apps, educational tools). Level 2 (Peer to peer): Interventions facilitated through peer support or group interactions without direct professional input. Level 3 (Minimal professional involvement): Interventions with limited guidance or support from health professionals (e.g., periodic check-ins). Level 4 (Ongoing real-time professional interaction): Interventions requiring sustained engagement with health professionals through real-time interactions (e.g., video consultations). Level 5 (Specialist real-time interaction): Interventions involving intensive or specialized real-time professional care (e.g., multidisciplinary team involvement). This framework provides a scalable approach to care, aligning intervention intensity with individual needs.

### 3.5. Technological Platform

Half of the virtual care solutions needed access to a website or computer (50.7%, *n* = 37). Close to half of the interventions involved teleconferencing (43.8%, *n* = 73). A third of the solutions (30%, *n* = 22) were offered via smartphones, and 6.8% (*n* = 5) were telephone based (e.g., used text messages or telephone calls). Over a third (31.5%, *n* = 23) used a combination of more than one technological platform (e.g., teleconferencing and a website).

### 3.6. Components of Virtual Care Solutions

Virtual care solutions were further classified based on the presence or absence of specific components identified from a previous systematic review focused on pediatric chronic pain [42], insights into relevant outcomes for caregivers from pediatric chronic pain clinical trials (PaedePPOC) [17], and input from our caregiver partners. Components are listed in Table 1 and include (1) psychological strategies, (2) physical/lifestyle strategies, (3) parenting strategies/skills, (4) informational needs, (5) social components, (6) practical support, (7) health professional communication, (8) user experience, and (9) target user.

#### 3.6.1. Psychological Strategies

Most (83.6%, *n* = 61) of the identified records involved psychological strategies to support caregivers. Of these, many included cognitive/thinking strategies (49.2%, *n* = 30), resilience building (46.0%, *n* = 28), and communication skills (37.7%, *n* = 23). Additional psychological components included mindfulness exercises (37.7%, *n* = 23), coping strategies (34.4%, *n* = 21), self-compassion practices (26.2%, *n* = 16), relaxation techniques (23.0%, *n* = 14), goal setting (18.0%, *n* = 11), and strategies with a spiritual element (14.8%, *n* = 9).

#### 3.6.2. Physical and Lifestyle Strategies

Only 15.1% (*n* = 11) of the identified studies included physical and lifestyle strategies. Of these, over half (54.5%, *n* = 6) offered information on diet and nutrition, sleep (54.5%, *n* = 6), and exercise and movement strategies (54.5%, *n* = 6).

#### 3.6.3. Parenting Strategies/Skills

One-third (31.5%, *n* = 23) of the studies provided parenting strategies/skills. Of these, almost half offered guidance on behavior management (43.5%, *n* = 10) or included the involvement of a parent coach (43.5%, *n* = 10). Others focused on communication skills, role modeling, promoting independence (17.4%, *n* = 4), and providing emotional support (13.0%, *n* = 3).

#### 3.6.4. Informational Needs

Almost half of the virtual care solutions supported caregivers’ informational needs (43.8%, *n* = 32). Of these, the majority (78.1%, *n* = 25) provided disease-specific or medical education, and over a third (37.5%, *n* = 12) provided information on symptom management. Only four (12.5%) studies focused on delivering information about financial assistance services, and six (18.4%) focused on information about accessing resources.

#### 3.6.5. Social and/or Family Engagement

Thirty-five percent (35.5%, *n* = 26) of records included social components to promote socialization, support, and/or family engagement. Of these, the majority (77%, *n* = 20) included peer mentorship and/or support groups. Only five (19%) focused on family support or counseling, and only one (3.8%) specifically focused on partner and sibling support.

#### 3.6.6. Practical Support

Only one virtual care solution (1.4%) included a component addressing practical support (i.e., respite support, housekeeping, meal preparation). This intervention focused on supporting caregivers at work and helping them to support their children at school.

#### 3.6.7. Healthcare Professional Communication

The majority (91.8% *n* = 67) of the records involved communication with healthcare professional(s). Thirteen reports (19.4%) incorporated real-time and asynchronous health professional communication. Most communication was in real time (64.2% *n* = 43), and over half was asynchronous (56.7% *n* = 38). Only one (1.5%) included computer-generated communication.

#### 3.6.8. Intended User

Most virtual care solutions targeted individual caregivers (71.2%, *n* = 52). Fifteen interventions (20.5%) offered content for groups of caregivers, and eleven (15.2%) offered paired interventions targeting both caregivers and their children.

#### 3.6.9. Quality of Evidence

Of the 73 records, the largest proportion (57.5%, *n* = 42) were rated as high quality, followed by moderate (37.1%, *n* = 27) quality. Three (4.1%, *n* = 3) were rated as low quality, and one (1.4%) was rated as critically low quality. Agreement among all raters was achieved for all quality ratings. Detailed quality ratings for each included record are provided in Supplementary Material [SC3].

### 3.7. Synthesis of Results

**Figure 4:**
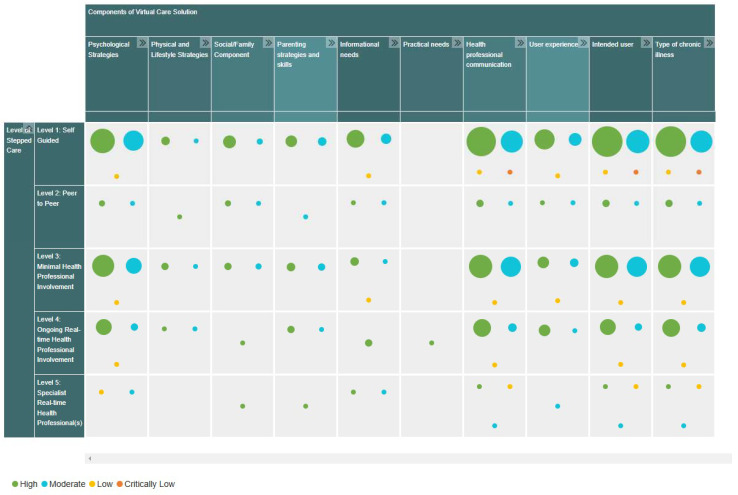
Static version of the Evidence and Gap Map (EGM) showing the number and quality of included studies across the stepped care continuum and virtual care components. The rows represent the five levels of the stepped care continuum (Figure 1), and the columns depict the higher-order components of virtual care solutions. Each cell indicates the intersection of these two dimensions, with study counts and quality ratings displayed. A small legend is included below to clarify the structure of the figure. For a more comprehensive and interactive exploration, the EGM is available online at https://lab.research.sickkids.ca/iouch/caregivers/, accessed on 18 December 2024, where users can apply filters and sort data to suit their needs.

Scoping review data were synthesized visually in the EGM using EPPI-Mapper 4.0 [55]. A static version of the EGM is presented in Figure 4, which provides an overview of the number and quality of included records. While Figure 4 serves as a summary, it has inherent limitations in conveying the full functionality and interactivity of the EGM. The rows represent the five levels of the stepped care continuum (Figure 1) and the columns capture the high-order components of each virtual care solution. Each EGM cell displays the number and quality of studies corresponding to the intersection of a specific level and virtual care component. For detailed exploration and enhanced usability, the interactive version of the EGM is publicly accessible online at https://lab.research.sickkids.ca/iouch/caregivers/, accessed on 18 December 2024. This interactive tool allows users to sort and filter data by codes such as evidence quality, target user, or technology platform and to view records collectively or individually.

Researchers interested in adding their work to the EGM are encouraged to contact the study team.

Small legend for Figure 4: Rows represent the five levels of the stepped care continuum (from self-guided interventions to specialized real-time interactions). Columns represent the higher-order components of virtual care solutions (e.g., psychological strategies, peer support, physical health components). Cells display the number of studies and their quality at the intersection of each level and component.

## 4. Discussion

This scoping review used an EGM as a novel synthesis tool to map the 73 scientific studies reporting virtual care solutions for caregivers of children with chronic illnesses across the stepped care continuum. The EGM identifies several areas of existing high-quality, evidence-based virtual care solutions and where few or no solutions exist. Most virtual care solutions primarily target caregivers of children with cancer, neurological disorders, and complex illnesses. Fewer address pediatric chronic pain or mental health conditions. Critically, very few of the identified interventions address what is emerging as one of the key drivers for pain outcomes in children: caregivers’ own mental health and chronic pain. This gap highlights the urgent need for interventions that not only improve caregiving behaviors but also directly address caregivers’ health and well-being. The publicly available EGM provides an accessible knowledge mobilization tool that enables diverse audiences, including patients, families, health professionals, and policymakers, to make evidence-informed decisions [40,41,42]. It also offers direction towards areas where new research, interventions, and policy are needed.

Several key points were identified by mapping current evidence for caregiver-targeted virtual care solutions in this format. We showed that psychological strategies were the most common component of virtual care interventions for caregivers. These included cognitive strategies, cognitive-behavioral techniques, positive reframing, goal setting, resilience building, coping skills, and mindfulness practices. The prioritization of psychological strategies reflects the significant mental health impact caregiving imposes [6,8,9] and their adaptability to virtual formats [21,56,57]. While effective, these strategies often focus on improving caregiver behaviors rather than directly addressing caregivers’ own mental and physical heath challenges.

Family-centered approaches, particularly those involving parent–child dyads, offer enhanced participation and improved health outcomes for both caregivers and children [21,58,59]. These approaches also promote healthier behaviors, indirectly improving physical health across generations. For example, a parent–child intervention focused on obesity prevention led to significant improvements in dietary intake and physical activity for both parents and children [60]. Similarly, a systematic review of combined interventions addressing physical activity, nutrition, and sleep among overweight families demonstrated notable health benefits across family members [61]. This highlights the potential of collaborative approaches to fostering holistic well-being. However, virtual care interventions must expand beyond psychological strategies to include practical supports tailored to caregivers’ diverse needs.

The EGM uncovered a notable gap: Few interventions directly addressed chronic pain in caregivers, as well as other physical health needs such as nutrition, or lifestyle factors, despite their relevance and proven benefits for caregiver populations [19,57,60,61,62]. This gap is especially concerning given that up to 50% of caregivers in tertiary pediatric pain clinics report experiencing chronic pain themselves [5]. This bidirectional relationship between caregiver mental health, chronic pain, and caregiving demands creates a cycle of stress and strain that adversely impacts both caregiver and child outcomes [4,5,6,7,8,15,16]. To break this cycle, holistic interventions that jointly address caregivers’ mental and physical health are required [14,15]. Family-centered interventions that integrate components that directly target caregivers’ pain management, nutrition, physical activity, and sleep hygiene and nutrition guidance into psychological interventions can provide comprehensive support for caregivers and their families. Policymakers and practitioners can leverage the EGM to prioritize the development and funding of interventions that address these gaps. For example, combining behavioral pain management strategies with practical support, such as respite services, meal preparation assistance, or flexible work arrangements, can reduce caregiver strain and improve their capacity to sustain caregiving roles. The EGM also highlights areas of minimal evidence, such as sibling-focused interventions or caregiver nutrition support, providing a roadmap for future research and resource allocation.

Although peer support was relatively common, our review showed a notable lack of virtual interventions specifically designed to offer structured sibling or family counseling. This gap is significant given the established benefits of family engagement in managing chronic conditions [23,25]. Engaging the entire family, including siblings and extended family, is essential to addressing the ripple effects of chronic pain on family dynamics and ensuring tailored support for all members. Only one intervention offered practical caregiver support, highlighting the need to address challenges like financial strain, employment conflicts, and limited access to respite services [6,7,8,34]. This lack of interventions likely reflects a broader gap in prioritizing these structural challenges within the development of virtual care solutions. Virtual care solutions must expand to include practical solutions alongside evidence-based mental health support. For example, integrating financial guidance, respite care, or sibling and family counseling into digital platforms could directly address the structural barriers caregivers face. These additions would not only reduce caregiving stress but also enhance engagement with psychological interventions. Policy- and decision-makers can use the EGM to guide these expansions, ensuring tailored interventions align with caregivers’ dual mental and practical needs.

Ensuring equitable access to virtual care interventions is critical. Strategies such as providing flexible, tailored services, leveraging digital tools, offering offline solutions (e.g., downloadable resources), implementing feedback systems, and engaging underserved communities in co-designing solutions enhance inclusivity and accessibility [63,64]. Practical support resources that offer respite and strategies for balancing responsibilities can improve caregiver capacity and improve child and family outcomes. To ensure these digital solutions address the unique needs and challenges of this population, it is critical to engage caregivers and other end-users in evaluating their relevance and applicability.

Recognizing this, our team collaborates with diverse international groups of caregivers, interdisciplinary clinicians, and researchers to assess how virtual care solutions can best support caregivers of children with chronic pain. A critical piece of our work has been the active engagement of caregiver partner advisors as equal, compensated, and integral members of the research team. Their lived experience and insights strengthened this scoping review, enhancing the interpretation of findings by highlighting gaps in existing solutions and emphasizing the practical and emotional dimensions of caregiving [43,44,45,46]. Caregiver partner advisors provided a critical lens for interpreting findings, highlighting practical gaps such as the lack of interventions targeting financial and respite care support, and emphasizing the need for holistic family-centered approaches. Their insights also informed the prioritization of gaps identified in the review, such as the lack of tailored, scalable solutions that address caregivers’ mental health and physical health needs. This integration of lived experience ensured the findings are reflective of real-world caregiver challenges and actionable for future research and innovation.

Findings from this scoping review provide the foundation for our ongoing work and highlight the priority for accessible, relevant, and inclusive digital solutions tailored to caregivers’ needs. By identifying gaps, such as a lack of scalable solutions that directly address caregiver mental health and chronic pain, this review emphasizes the importance of holistic approaches. The EGM serves as a critical tool for guiding future research and intervention design, enabling policymakers, practitioners, and researchers to prioritize areas with limited evidence, allocate resources effectively, and develop tailored, scalable interventions that address caregivers’ mental, physical, and practical needs. These insights will directly inform our co-designing of a digital platform to deliver rapid, accessible, and tailored support for caregivers of children with chronic pain. Our collaborative and co-creative approach aims to address the global needs of families impacted by chronic pain while advancing best practices for meaningful partnerships with people with lived and living experiences in research.

### Limitations

Although this multimethod scoping review is comprehensive, several limitations should be acknowledged. This review included only English-language studies, potentially introducing language bias and limiting the usefulness of findings to people who do not speak English. This exclusion may have resulted in the omission of culturally specific interventions or innovative solutions developed in non-English-speaking regions. Future reviews could address this gap by including studies in multiple languages, leveraging translation services to enhance inclusivity.

Although a call for innovations was conducted, a comprehensive search of grey literature (i.e., conference abstracts, dissertations, unpublished studies) was not included. This limitation may have restricted the identification of emerging virtual care solutions, particularly those still in early stages of development or outside traditional academic publishing channels. Future reviews could incorporate grey literature to capture these innovations and provide a more complete picture of the landscape of virtual care solutions. The broadened scope to include virtual care for caregivers of children with chronic illnesses in general, rather than solely chronic pain, may have reduced specificity in addressing the unique challenges faced by families managing pediatric chronic pain. The review identified a notable gap in practical, hands-on support solutions, such as respite care, financial guidance, or employment assistance. This suggests that essential caregiver needs may be underrepresented in the current literature.

## 5. Conclusions

This scoping review and EGM highlight the urgent need for scalable, accessible, and evidence-based virtual care solutions to support caregivers of children with chronic illnesses, including chronic pain. While existing digital solutions often target psychological strategies, significant gaps remain in addressing caregivers’ mental and physical health, practical resources, family-centered counseling, and lifestyle support. The lack of tailored solutions for marginalized and underserved populations underscores the need to prioritize health equity in virtual care design and delivery. Partnering with caregivers in the co-design of digital solutions ensures that their lived experience shapes solutions that holistically address the complexities of caregiving.

This work emphasized the importance of integrating caregiver support into family-oriented care and leveraging technologies to scale accessible, equitable solutions. Health professionals play an integral role in advocating for and delivering tailored digital solutions that address the diverse needs of families. The EGM serves as a valuable tool for synthesizing evidence, identifying gaps, and guiding decision-making for families, clinicians, researchers, and policymakers. Future efforts should focus on developing and evaluating virtual care solutions that comprehensively support caregivers’ mental, physical, and practical needs, fostering resilience and improving outcomes for families managing chronic pediatric pain.

## Figures and Tables

**Table 1:** Coded components of virtual care solutions included in Evidence and Gap Map.

Higher-Order Code	Lower-Order Codes
Psychological strategies	Relaxation Mindfulness Resilience (e.g., psychoeducation) Spiritual Thinking strategies (e.g., cognitive strategies, reframing, emotional regulation) Communication skills (e.g., problem-solving skills) Resilience Coping Goal setting Self-compassion
Physical and lifestyle strategies	Sleep hygiene (e.g., strategies that support improved sleep) Diet/nutrition Exercise/movement Health check-ups/monitoring (e.g., screening own health—not the child) Work–life balance
Social component	Peer mentorship/support groups/programs Familial support/counseling Partner and sibling relationship/support Other social support
Health professional communication	Asynchronous (i.e., not delivered or received in real time but rather on each participant’s own time) Real-time (i.e., synchronous) Computer generated (i.e., automated appointment/medication/follow-up reminders)
User experience (i.e., evidence or data addressing)	Functionality Usability Accessibility (as per Web Content Accessibility Guidelines 2.0) Acceptability Feasibility Customizability
Intended user	Individual (e.g., parent of a youth with chronic pain) Paired caregiver–youth (e.g., joint intervention) Group (e.g., multiple parents) Not reported (e.g., information not available) Health professionals
Type of chronic illness	Cancer/oncology Diabetes Developmental (e.g., ASD, cerebral palsy, intellectual disabilities) Learning and attentional (i.e., ADHD) Chronic pain Gastrointestinal (e.g., inflammatory bowel disease) ‘Chronic illness’ (general) Neurological (e.g., traumatic brain injury) Other
Parenting strategies/skills	Parent support/parenting coach (i.e., 1:1 parent support programs/skills) Behavior management (i.e., parenting intervention targeting challenging behaviors in children) Communication skills (e.g., active listening, positive reinforcement) Providing emotional support (e.g., empathy, attachment building) Providing teaching and learning support (i.e., role modeling, promoting independence)
Informational needs	Disease/medical education Symptom management Resource access (i.e., where/how to access resources and services) Other professionals (i.e., where/how to access other healthcare professionals/services or groups) Financial assistant services (i.e., how to access financial support)
Practical needs	Work/Employment (i.e., remote work options, flexible work hours) Housekeeping support (i.e., help with cleaning tasks, laundry, etc.) Cost of care (i.e., rebates, refunds, discounts, allowances, subsidies for care) Financial support/assistance (i.e., other financial support such as disability schemes, etc.) Childminding services (i.e., nanny services, daycare, home-based child minding, before- and after-school care) Respite care (i.e., in-home respite care, emergency respite care)

## Data Availability

All data are included in the article or in the tables.

## Supplementary Materials

The following supporting information can be downloaded at https://www.mdpi.com/article/10.3390/children12010077/s1. SC T1: [PDF] Sample search strategy for scientific literature search, SC T2: [Excel] Records in the Evidence and Gap Map, SC T3: [Excel] Mixed Methods Appraisal Tool (MMAT) quality ratings for scientific records in the Evidence and Gap Map.
