# Supplementary material for: Mapping the Gaps: A Scoping Review of Virtual Care Solutions for Caregivers of Children with Chronic Illnesses
Source: Children (Basel). 2025 Jan 10;12(1):77. doi: 10.3390/children12010077 (PMC11763621; doi:10.3390/children12010077)
Supplement: Supplementary file 1 [file children-12-00077-s001.zip › SCT1 CINAHL Database search.pdf]

EVIDENCE AND GAP MAP OF VIRTUAL SOLUTIONS FOR YOUTH WITH PAIN AND THEIR FAMILIES

Supplementary Table 1. Sample search strategy for scientific literature search

CINAHL (EBSCO) SEARCH

1. (MH "Telehealth") OR (MH "Remote Consultation") OR (MH "Telemedicine") OR (MH "Telerehabilitation") OR (MH "Telenursing") OR (MH "Telenutrition") OR (MH "Telepsychiatry")
2. TI (telehealth* or tele-health* or telemedicin* or tele-medicin* or telenur* or tele-nur* or teleconsult* or tele-consult* or rtelehealth* or rtele-health* or telemonitor* or tele-monitor* or phone* or telepsychiatr* or tele-psychiatr* or teletherap* or tele-therap*) OR AB (telehealth* or tele-health* or telemedicin* or tele-medicin* or telenur* or tele-nur* or teleconsult* or tele-consult* or rtelehealth* or rtele-health* or telemonitor* or tele-monitor* or phone* or telepsychiatr* or tele-psychiatr* or teletherap* or tele-therap*)
3. TI ((virtual* and (care or medicine or health*)) not virtual-reality) OR AB ((virtual* and (care or medicine or health*)) not virtual-reality)
4. (MH "Internet+")
5. TI (digital or online* or internet) OR AB (digital or online* or internet)
6. TI (videoconferenc* or video-conferenc* or webbased or web-based or web-page* or webpage* or web-site* or website* or webex or whatsapp or www) OR AB (videoconferenc* or video-conferenc* or webbased or web-based or web-page* or webpage* or web-site* or website* or webex or whatsapp or www)
7. TI (e-health* or ehealth*) OR AB (e-health* or ehealth*)
8. TI (cellphone* or smartphone*) OR AB (cellphone* or smartphone*)
9. TI (mhealth* or m-health* or mobile-health*) OR AB (mhealth* or m-health* or mobile-health*)
10. (MH "Mobile Applications")
11. TI (app or apps) OR AB (app or apps)
12. (MH "Telecommunications") OR (MH "Electronic Bulletin Boards") OR (MH "Email") OR (MH "Instant Messaging") OR (MH "Interactive Voice Response Systems") OR (MH "Text Messaging")
13. TI (Teleconferenc* or Tele-conferenc* or Telecommunication* or Tele-communication*) OR AB (Teleconferenc* or Tele-conferenc* or Telecommunication* or Tele-communication*)
14. TI (Text-messag* or SMS or email* or e-mail*) OR AB (Text-messag* or SMS or email* or e-mail*)
15. TI (e-resource* or e-support* or eresource* or esupport*) OR AB (e-resource* or e-support* or eresource* or esupport*)
16. (MH "Communications Software") OR (MH "Client-Server Application") OR (MH "Patient Portals") OR (MH "Mobile Applications") OR (MH "Multimedia")
17. (MH "Software")

18. TI (computer* or electronic* or remote or software*) OR AB (computer* or electronic* or remote or software*)
19. TI (facebook or ICT or ICTs or Information-and-communications-technolog* or instant-messag* or Chat or chats or chatroom*) OR AB (facebook or ICT or ICTs or Information-and-communications-technolog* or instant-messag* or Chat or chats or chatroom*)
20. TI psychological OR AB psychological
21. 1 or 2 or 3 or 4 or 5 or 6 or 7 or 8 or 9 or 10 or 11 or 12 or 13 or 14 or 15 or 16 or 17 or 18 or 19 or 20
22. (MH "Parents+")
23. (MH "Caregivers")
24. (MH "Guardianship, Legal+")
25. (MH "Family+")
26. TI (parent* or father* or mother* or paternal* or maternal* or caregiver* or care-giver* or caretaker* or care-taker* or guardian* or family or families) OR AB (parent* or father* or mother* or paternal* or maternal* or caregiver* or care-giver* or caretaker* or care-taker* or guardian* or family or families)
27. 22 or 23 or 24 or 25 or 26
28. TI (newborn* or new-born* or baby or babies or neonat* or neo-nat* or infan* or toddler* or pre-schooler* or preschooler* or kinder or kinders or kindergarten* or kinder-aged or boy or boys or girl or girls or child* or pediatric* or paediatric* or school-age* or schoolage* or schoolchild* or schoolgirl* or schoolboy* or minor or minors or adolescen* or youth or youths or teen or teens or teenage*) OR AB (newborn* or new-born* or baby or babies or neonat* or neo-nat* or infan* or toddler* or pre-schooler* or preschooler* or kinder or kinders or kindergarten* or kinder-aged or boy or boys or girl or girls or child* or pediatric* or paediatric* or school-age* or schoolage* or schoolchild* or schoolgirl* or schoolboy* or minor or minors or adolescen* or youth or youths or teen or teens or teenage*)
29. TI (children-with-special-healthcare-need* or children-with-special-health-need* or children-with-special-need* or CSHCN) OR AB (children-with-special-healthcare-need* or children-with-special-health-need* or children-with-special-need* or CSHCN)
30. TI (complex* N1 (medical* or care* or health* or need*)) OR AB (complex* N1 (medical* or care* or health* or need*))
31. TI medical* N1 fragil* OR AB medical* N1 fragil*
32. (MH "Pain+")
33. (MH "Chronic Disease+")
34. TI ((long-term or chronic* or activity-limiting or persistent) and (disorder* or condition* or ill* or sick*)) OR AB ((long-term or chronic* or activity-limiting or persistent) and (disorder* or condition* or ill* or sick*))
35. (MH "Rheumatic Diseases+")
36. TI (pain or pains) OR AB (pain or pains)
37. (MH "Complex Regional Pain Syndromes+")
38. (MH "Inflammatory Bowel Diseases+")

39. (MH "Menstruation Disorders+")
40. (MH "Diabetes Mellitus+")
41. (MH "Neoplasms+")
42. (MH "Genital Diseases, Female+")
43. (MH "Anemia, Sickle Cell+")
44. (MH "Skin Diseases+")
45. TI headache* OR AB headache*
46. TI (endometriosis or endometrioses) OR AB (endometriosis or endometrioses)
47. TI dysmenorrh* OR AB dysmenorrh*
48. TI (rheumat* or arthriti* or fibromyalgi*) OR AB (rheumat* or arthriti* or fibromyalgi*)
49. TI (cancer* or neoplas* or tumor* or tumour* or malignan* or carcinoma*) OR AB (cancer* or neoplas* or tumor* or tumour* or malignan* or carcinoma*)
50. TI ((gynecologic* or gynaecologic*) N4 (disease* or disorder*)) OR AB ((gynecologic* or gynaecologic*) N4 (disease* or disorder*))
51. TI (((skin or dermal* or dermatolog*) N4 (disease* or disorder*)) or eczema*)) OR AB (((skin or dermal* or dermatolog*) N4 (disease* or disorder*)) or eczema*))
52. TI ((sickle-cell N4 (disease* or disorder* or anemia*) or SCD) OR AB ((sickle-cell N4 (disease* or disorder* or anemia*) or SCD)
53. TI ((bowel* N4 inflammatory N4 (condition* or disease* or illness* or syndrome*)) or IBS or IBD) OR AB ((bowel* N4 inflammatory N4 (condition* or disease* or illness* or syndrome*)) or IBS or IBD)
54. TI asthma* OR AB asthma*
55. TI diabet* OR AB diabet*
56. (MH "Brain Injuries+")
57. TI (brain N4 (trauma* or injur* or TBI or TBIs)) OR AB (brain N4 (trauma* or injur* or TBI or TBIs))
58. 30 or 31 or 32 or 33 or 34 or 35 or 36 or 37 or 38 or 39 or 40 or 41 or 42 or 43 or 44 or 45 or 46 or 47 or 48 or 49 or 50 or 51 or 52 or 53 or 54 or 55 or 56 or 57
59. Random* OR placebo* OR therap* OR trial* OR groups OR double-blind OR single-blind OR research design OR practice guideline*
60. intervention* or evaluat* or program* or effective* or efficac* or chang* or outcome* or success* or fail* or high* or low* or increase* or reduc* or harm* or implement* or treat* or therap*
61. 59 or 60
62. 21 and 27 and 28 and 58 and 61
63. 21 and 27 and 29 and 61
64. 62 or 63
